# Overview of availability of harm reduction interventions in European prisons

**DOI:** 10.1093/eurpub/ckac129.386

**Published:** 2022-10-25

**Authors:** L Montanari, S Mazzilli, A Tarján, I Hasselberg, W Hall, L Vandam, A Vernooij, H Stöver

**Affiliations:** 1 Public Health Unit, EMCDDA, Lisbon, Portugal; 2 Department of Translational Research in Medicine, University of Pisa, Pisa, Italy; 3 Scuola Normale Superiore, Pisa, Italy; 4 Hungarian Reitox National Focal Point, Budapest, Hungary; 5 Centre for Research in Anthropology, University of Minho, Braga, Portugal; 6 National Centre for Youth Substance Use, The University of Queensland, Brisbane, Australia; 7 Institute of Addiction Research, University of Applied Sciences, Frankfurt am Main, Germany

## Abstract

**Introduction:**

Prisons are high-risk environments for the transmission of drug related infections, due to over-incarceration of people who inject drugs; often inadequate healthcare, substandard prison conditions; and others. An overview of the availability and coverage of prison-based harm reduction interventions in Europe is presented.

**Methods:**

National Focal Points of the EMCDDA (30) collected 2019 data, which were integrated with findings from the European funded project HA-REACT (Joint Action on HIV and Co-infection Prevention and Harm Reduction).

**Results:**

Prison based harm reduction interventions are available in European countries, but only few of them are available in most countries and often with a low coverage (e.g. less than 10% of prison population in Opioid Substitution Treatment (OST) in most countries). Interventions available in most countries (20 or more) include: HIV, HBV, HCV testing (29), OST continued from community (29), Referral to HIV treatment upon release (28), HIV treatment (27), Referral to HCV treatment upon release (25), HCV antiviral treatment (25), Testing for TB (23), HBV antiviral therapy (25), OST initiated in prison (22), Treatment for TB (21), Vaccination for HBV (20). Interventions available in 10 to 19 countries are: condom distribution (19), OST (re)initiated before release (17), prison/community guidelines for implementation of OST (13). Interventions provided in < 10 countries include: distribution of disinfectant (9), condom with lubricant (9), take-home naloxone (5), needles and syringes programs (3).

**Conclusions:**

Compared to the community, the availability and coverage of harm reduction interventions in European prisons are limited and large information gaps exist. Scaling up harm reduction in prison can achieve important individual and public-health benefits.

